# 

**Published:** 1894-10

**Authors:** 


### CONTENTS FOR OCTOBER, 1894.

ORIGINAL COMMUNICATIONS. PAGE.

The Causes of Hernia—With a New Theory. By BYRON ROBINSON .......... 129
The Treatment of Neurasthenia. By WILLIAM C. KRAUSS, M. D .......... 132
Kaposi's Clinic. Interesting Dermatological Cases. By FRANK J. THORNBURY, M. D... 136
Hypertrophy of the Follicular Glands at the Base of the Tongue. By HORACE CLARK, M. D .......... 143

TRANSLATION.

Sarcoma of the Tongue Treated with Pyoktanin. By L. G. SELLSTEDT, Esq.......... 148

SELECTION.

Revival of Symphyseotomy .......... 152

THERAPEUTIC NOTES.

Metric Prescriptions .......... 160

CORRESPONDENCE.

Philadelphia Letter .......... 162
Multiple Papillomes of the Larynx .......... 165

EDITORIAL.

American Association of Obstetricians and Gynecologists .......... 166
Topics of the Month .......... 168

Personal .......... 173
Obituary .......... 174
Society Meetings .......... 175
Hospital Notes .......... 176
Book Reviews .......... 177
Literary Notes .......... 186
Miscellany .......... 186

